# Urgent COVID-19 Vaccination of Healthcare Workers via a Quality Improvement Initiative

**DOI:** 10.1097/pq9.0000000000000532

**Published:** 2021-11-29

**Authors:** Shannon H. Baumer-Mouradian, Stacey Collins, Thomas Lausten, Cecile Pohl, Mary Sisney, Smriti Khare, Megan Ose, Jennifer Roe, Chelsi Reilly, Michael Gutzeit

**Affiliations:** From the * Department of Pediatrics, Medical College of Wisconsin, Milwaukee, Wis.; † Children’s Hospital of Wisconsin, Milwaukee, Wis.

## Abstract

The COVID-19 pandemic has posed a significant threat to US healthcare workers’ mental and physical health. The US Food and Drug Administration approved the first mRNA COVID-19 vaccine for Emergency Use Authorization on December 11, 2020. High-risk healthcare workers were determined to be Phase 1a. Goal: Complete the two-dose vaccine series in all interested phase 1a staff immediately after the COVID-19 vaccine was available and distributed to our institution, December 14, 2020.

**Methods:**

A multidisciplinary team involving key stakeholders performed process mapping to develop four key drivers for vaccination success: rapid vaccine procurement, proper storage and handling, well-defined vaccine administration and follow-up plan, and system preparation. We tested interventions using plan-do-study-act cycles. We included employees and providers with direct patient care responsibilities, age 18 years or older, employed at the children’s health system, or the affiliated academic medical center. We examined the total number of dose 1 and dose 2 vaccines administered for our primary outcome, and the balancing measure included the percent of wasted vaccines.

**Results:**

Three thousand nine-hundred twenty-one healthcare personnel completed the survey, and 73% reported intent to receive the COVID-19 vaccine immediately or at a later time. After 57 clinic days, we vaccinated 83% (n = 5,231) of healthcare personnel at our institution, and 99% completed the two-dose series. Due to surplus vaccines, we vaccinated an additional 1,258 community members with 99% completion of the two-dose series. Vaccine waste was minimal 0.1%.

**Conclusion:**

We describe the development and implementation of a successful COVID-19 employee and community vaccination program.

## Abstract 2

Supplemental Digital Content is available in the text.

## INTRODUCTION

The coronavirus disease 2019 (COVID-19) pandemic has posed a significant threat to the US healthcare workers. As of April 2021, over 3,600 healthcare workers died within the first year of the Pandemic due to COVID-19-related illness.^1^ Additionally, healthcare workers’ mental health has been heavily impacted, with 23% experiencing anxiety and 23% with depression due to concerns for their safety and the fear of transmitting the virus to their families.^2^

On December 11, 2020, the Food and Drug Administration approved the first severe acute respiratory syndrome coronavirus (SARS-CoV-2) vaccine for Emergency Use Authorization to prevent COVID-19 in patients 18 years and older.^3^ Phase 3 clinical trials demonstrated the vaccine was 95% effective in preventing COVID-19 symptoms.^4^ This mRNA vaccine required intramuscular administration of a two-dose series three weeks apart as well as 70°F storage.^5^

The Centers for Disease Control and Prevention (CDC) announced that vaccine demand would likely exceed available vaccines during the first months of distribution; therefore, the Advisory Committee on Immunization Practices prioritized healthcare workers and residents in long-term care facilities as Phase 1a, first priority.^6^ At our institution, we specifically followed guidelines for vaccine distribution outlined by the Wisconsin Department of Health Services (DHS) State Disaster Medicine Committee.^7^ Our institution had approximately 6,300 healthcare personnel and approximately 4,000 meeting Phase 1a criteria.

## OBJECTIVE

We set a specific aim to complete the two-dose vaccine series for all interested Phase 1a staff immediately after the COVID-19 vaccine was available and distributed to our institution, December 14, 2020.

## METHODS

### Context

This children’s health system is a free-standing, not-for-profit pediatric academic center located in Milwaukee, Wis., and comprises a tertiary care hospital and primary and specialty pediatric care services. In November 2020, a multidisciplinary “vaccine team,” including hospital leadership, employee health and wellness, nursing and pharmacy leadership, and a provider, formed to develop and implement a COVID-19 vaccination program for all employees. Before this project, vaccine team members oversaw a mandatory employee influenza vaccination program annually with the capacity to vaccinate up to 100 individuals per day. Vaccine team members monitored vaccine inventory and administration using an electronic database. Additionally, our pharmacy had an −80°F freezer with the capacity to hold 10,000 vaccine doses.

### Interventions

**Fig. 1.**
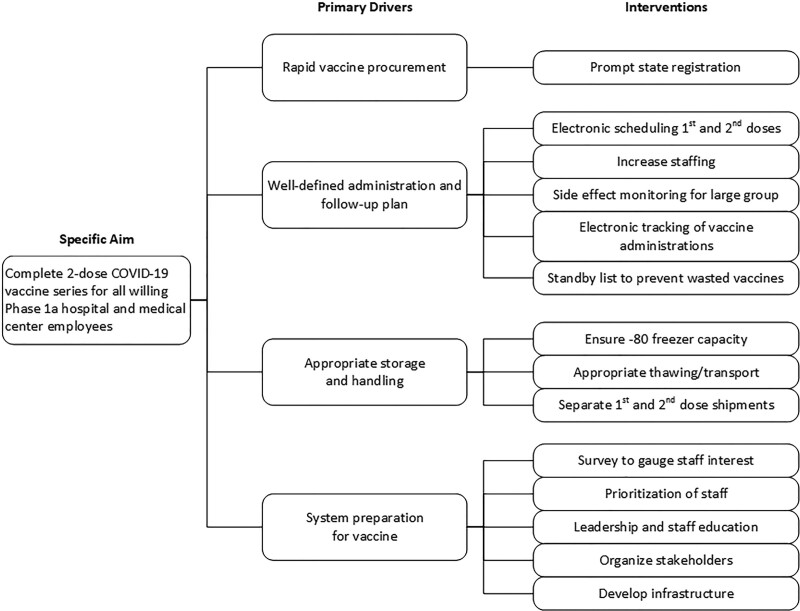
Key driver diagram depicting the four key drivers and proposed interventions.

The COVID-19 vaccine planning process was unique as most of the planning occurred before specific knowledge of vaccine type, storage requirements, number of doses in the series, available supply, or employee vaccine demand. To prepare for immediate vaccine distribution once available, stakeholder analysis and failure modes and effects analysis led us to develop a flexible process that satisfied the requirements for multiple vaccine candidates. Four key drivers included: rapid vaccine procurement, well-defined vaccine administration and follow-up plan, proper storage and handling, and system preparation (Fig. 1). The target population included phase 1a staff/providers with direct patient care responsibilities, age 18 years or older, employed at the children’s health system or the affiliated academic medical center. For this process, we categorized numerous interventions around four key drivers.

### Rapid Vaccine Procurement

Our team followed Wisconsin DHS requirements to become a COVID-19 vaccine distribution center, including completing a provider enrollment form, obtaining access to the Wisconsin Immunization Registry (WIR), and participating in vaccine storage, handling, and administration training. In addition, hospital leadership provided documentation of vaccination capacity and the number of vaccine-eligible employees to DHS. As a result, on December 14, 2020, our institution was one of the first locally to become an authorized COVID-19 vaccine distribution center. The following day, 2,975 COVID-19 vaccines arrived in ultracold storage. The pharmacy also submitted weekly requests to acquire additional vaccines.

### Storage and Handling

Storage and handling of the COVID-19 vaccine were unique from prior influenza vaccination efforts. (1) The need for ultracold storage necessitated storage of vaccines in the central hospital pharmacy. (2) Daily thawing, dilution, and distribution to vaccine clinic required trained pharmacy and support staff. (3) DHS mandated strict monitoring for vaccine waste as well as possible diversion of vaccine. Upon vaccine shipment arrival, four pharmacy staff would redistribute large volume trays of vaccines into plastic bags in five-vial aliquots and store them in the hospital −80°F freezer. The process required 5 minutes for total transfer time. Storing vials in clusters of five allowed the team to access vaccines quickly without thawing a large volume tray.

On the day of a vaccine clinic, employee health and wellness requested a specific number of vaccines based on scheduling needs. Pharmacy prepared vaccines by thawing and diluting them according to manufacturer guidelines.^5^ To prevent expiration of thawed doses during the 6-hour vaccine clinic, pharmacy prepared vaccines in two batches, 75% of requested doses in the morning and 25% in the afternoon. The pharmacy utilized freezer locks, continuous video monitoring of the vaccine storage facility, and security escorts for vaccine transportation to prevent vaccine diversion—temperature sensors on freezers alarmed to prevent vaccine loss due to equipment malfunction. Furthermore, pharmacy leadership received an automated page for any 10° temperature fluctuation. A backup −80°F freezer was also available.

The state health department distributed dose 1 and dose 2 vaccines separately. Pharmacy distinguished doses 1 and 2 vaccine shipments by storing them on separate shelves in the −80°F freezer.

### Well-defined Administration and Follow-up Plan

#### Scheduling Dose 1

**Fig. 2.**
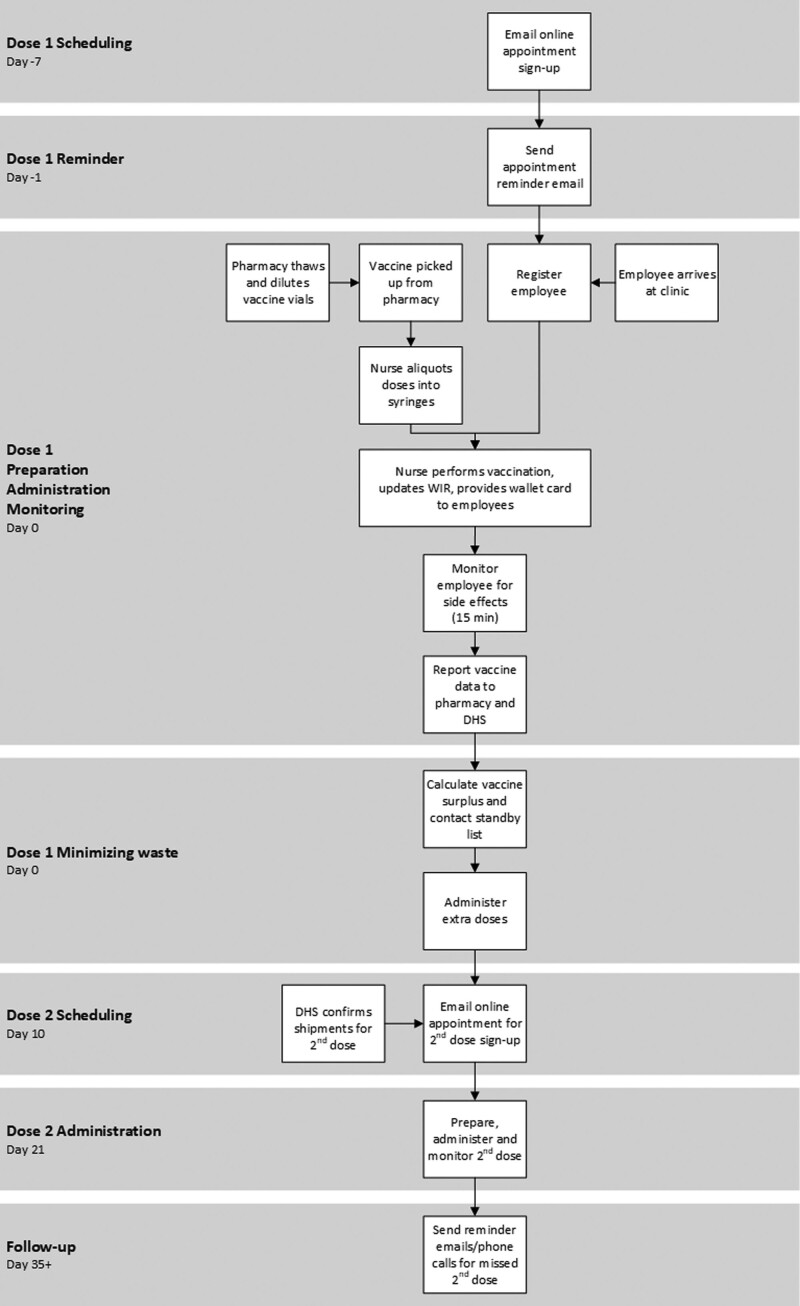
Process map summarizing the nursing vaccination workflow for scheduling, administration, and monitoring dose 1 and dose 2 vaccines over time.

Scheduling vaccine administration needed to be exact to eliminate any wasted doses. The team utilized an online scheduling platform emailed to employees, allowing them to self-enroll and choose a convenient vaccination slot. Initially, the vaccine team scheduled 300 dose 1 appointments, 1–5 days/week, and sent a reminder email to staff 24 hours before the appointment time. The vaccine team deferred dose 2 scheduling until the state had confirmed dose 2 shipments (Fig. 2).

#### Dose 1 Administration

Each clinic day, vaccine vials arrived at the clinic thawed and diluted. The assigned nurse would draw up five to seven 0.3-ml vaccine doses from each vial into syringes using one of two needle sizes depending on the patient’s body habitus. On employee arrival to the clinic, socially distanced markings allowed employees to register, wait in line, and enter one of 10 stations in a central vaccination room while maintaining 6 ft of distance. At each station, the nurse vaccinator would confirm vaccine eligibility, request a vaccine with the appropriately sized needle, receive a vaccine from the runner, perform vaccination, update the electronic health record and WIR, and provide a wallet card to the employee. To accommodate a 15-minute observation time per individual, 2–3 staff members monitored all vaccinated employees in a single large room with socially distanced chairs to assess for side effects. In addition, employee Health and Wellness trained nurses to identify signs of anaphylaxis and to administer Epi-pens when necessary.

#### Minimize Waste

Each day, employee health and wellness staff documented surplus vaccine doses due to no-shows or variation in the number of doses drawn per vial. To eliminate the need to discard these extra doses, vaccine clinic leadership reserved the final hour of the clinic for calling and administering vaccines to additional eligible employees. In addition, the clinic leadership trialed multiple PDSA cycles to finalize this end-of-the-day process and limit waste.

#### Documentation

Administrative support recorded vaccinations into the WIR. To maintain our status as a vaccine distribution center, pharmacy leadership submitted daily inventory reports documenting the number of vials used, doses administered, and doses wasted to DHS.

#### Scheduling Dose 2

To ensure dose 2 completion, clinic staff provided each vaccinated employee a wallet card that included the date of the dose 1 vaccine and the earliest date for dose 2. Two weeks after dose 1, following DHS confirmation of dose 2 shipments, employees received an email invitation to self-enroll for dose 2. In addition, the team tracked the successful completion of dose 2 vaccines, sent reminder emails, queried the WIR to determine if the individual received the dose elsewhere, and called individuals missing dose 2.

#### Dose 2 Administration

Dose 2 administration mimicked dose 1; however, dose 1 and dose 2 clinics were held on alternate days to avoid confusion between the different vaccine stocks.

### System Preparation

Due to significant differences in the COVID-19 vaccination program and prior employee vaccination efforts (influenza), significant system changes were necessary to prepare for vaccination, including gaging employee interest, staff prioritization for vaccination, vaccine clinic staffing, and vaccine education. Gaging employee interest in the vaccine was essential to estimate vaccine stock as well as vaccine clinic capacity. The vaccine team developed and administered an employee survey to quantify intent for vaccination, determine risk factors for COVID-19 (role in the organization and underlying medical conditions) and identify additional information that might inform decision-making. Given the concern for limited vaccine supply, hospital leadership prioritized employees using DHS guidelines. Individuals with direct patient care responsibilities and those with the potential to be around COVID-19 positive or unknown patients received high priority.^7^

To accommodate the need to administer 300 vaccines per clinic day, additional staff members from our “Labor Pool” received training for the vaccine clinic. The “Labor Pool,” developed during the COVID-19 Pandemic, included employees looking to gain extra hours by taking on new roles. Training included proper use of personal protective equipment, social distancing, and vaccine administration/documentation. Given the “newness” of the vaccine, the vaccine team considered education a critical step to address vaccine hesitancy. Therefore, we notified section leaders and asked them to determine the number of employees willing to be vaccinated in each section. In addition, employees received education via email announcements and institution-wide town hall meetings.

### Study of the Intervention

The vaccination team identified two problems leading to wasted vaccine doses. First, there was a large variation in the number of extra vaccine doses per day due to no-shows and variability in the number of doses drawn per vial. Second, the vaccination team scrambled to fill open vaccination slots and avoid wasted doses at the end of the clinic day. Therefore, the team needed a predictable number of extra doses and a simplified method to administer extra doses. These concerns led to two interventions:

1. Identifying Dose Management Nurses: To reduce variation in the number of doses drawn per vial, one or two “dose management nurses” were given the sole role to draw up vaccines each day, leading to six predictable doses per vial. We revised the schedule to accommodate six appointments per available vial (330 appointments per day).
2. Creation of a Standby list: We developed a standby list of unvaccinated individuals, meeting CDC guidelines, to fill vaccination slots on short notice.^6^ Additionally, we considered any individual arriving 30 minutes after their scheduled vaccine appointment to be a no-show. Following these two interventions, the predictability of extra doses improved, allowing more time to schedule individuals from the standby list. The vaccine team reserved the final hour of the clinic to administer extra vaccine doses to standby list members.

Finally, within 2 weeks of program initiation, most interested employees had been vaccinated, and vaccine clinics were no longer running at capacity. Therefore, our health system chose to support our local community by expanding the vaccine program to all eligible community members per CDC guidelines.

### Measures

The primary outcome was the total number of dose 1 and dose 2 vaccines administered and the percentage of vaccinated hospital/medical center employees. The balancing measure included the percent of wasted vaccines and adverse side effects.

### Analysis

We used descriptive statistics to describe the demographics and clinical/nonclinical roles of vaccinated individuals. A run chart demonstrates the cumulative number of dose 1 and dose 2 vaccines given each clinic day.

### Ethical Considerations

Leadership members of the quality committee at our hospital reviewed this project and determined it to be quality improvement work; therefore, it did not meet local requirements for Institutional Review Board review.

## RESULTS

Three thousand nine-hundred twenty-one employees at our institution participated in an anonymous survey in January 2020. Survey questions were optional; therefore, the number of responses per question varied from 3,921 to 1,722 (Table 1). Regarding risk for COVID-19, 72% self-reported they worked in a role that provided direct patient care, and 23% reported a history of a medical condition determined to be high risk for COVID-19 per CDC guidelines. Fifty-five percent reported an intent to receive the COVID-19 vaccine as soon as it was available, and an additional 18% reported they would consider receiving the vaccine at a later time. In response to, “Which of the following would help move your intention toward definitely receiving the COVID-19 vaccine?” the most common answers were more information about the vaccine’s safety and effectiveness (**Table 1, Supplemental Digital Content 1**, which describes employee survey responses indicating the need for further information to inform vaccine decision-making, http://links.lww.com/PQ9/A358). Although most of the respondents worked at the main hospital campus, of the 32% working at remote sites, 87% reported they would be willing to travel to receive the vaccine (~30 minutes) (Table 1).

**Table 1.** Employee Survey Results for 3,921 Employees

Survey Question	n	Yes (%)	No (%)	No, but at a Later Time	Unsure
Knowing a COVID-19 vaccine is on the way provides some relief or hope for the future.	3,921	3,020 (77%)	364 (9%)		537 (14%)
Work in a role that provides direct patient/client care/interaction.	3,914	2,820 (72%)	1094 (28%)		
Have an identified high-risk medical condition per CDC guidelines	3,921	910 (23%)	2,851 (73%)		160 (4%)
Intend to receive COVID-19 vaccine as soon as it is available	3,904	2,164 (55%)	516 (13%)	698 (18%)	526 (14%)
If you do not work at the main campus, are you willing to travel to receive the vaccine	696	609 (87%)	27 (4%)		60 (9%)

Our institution hosted 57 COVID-19 vaccine clinic days between December 17, 2020, and June 26, 2021. We administered 12,892 vaccines to 6,489 individuals. Demographic characteristics and clinical roles for vaccinated individuals are listed in (Table 2). We offered first priority for vaccine sign-up to all phase 1a staff; however, we expanded the vaccination program to include all hospital staff due to surplus vaccine. As a result, 5,231 hospital/academic center employees (83% of all staff) received dose 1 of the COVID-19 vaccine, and 5,168 (98.8%) of these employees received dose 2. In addition, seven of these individuals received dose 2 at an outside location.

**Table 2.** Demographic Characteristics and Clinical/Nonclinical Roles of Individuals Vaccinated via This COVID-19 Vaccination Program

	Black or African American, n = 305 (%)	White or Caucasian, n = 4,878 (%)	American Indian or Alaskan Native, n = 29 (%)	Asian, n = 244 (%)	Multiracial, n = 59 (%)	Other, n = 110 (%)	Unknown, n = 864 (%)	Total, n = 6,489
Community medical providers	14	219	1	11	3	1	57	306
Community member/other	30	251	3	5	6	0	63	358
Household contacts	23	305	1	15	3	0	76	423
Hospital and Academic Center	233	3,981	24	213	44	109	627	5,231
Retired	5	122	0	0	3	0	41	171
Total	305 (4.7)	4,878 (75.2)	29 (0.4)	244 (3.8)	59 (0.9)	110 (1.7)	864 (13.3)	6,489

Following the establishment of this vaccination program, our institution accepted the charge of expanding our vaccination program to offer vaccines to community members per CDC guidelines.^6^ One thousand two-hundred fifty-eight nonemployees received dose 1, and 1,250 (99.4%) completed dose 2. Vaccinated nonemployees included community medical providers (24%), community members over 65 years old & community hospital contractors (28%), household contacts (34%), and retired employees (14%).

**Fig. 3.**
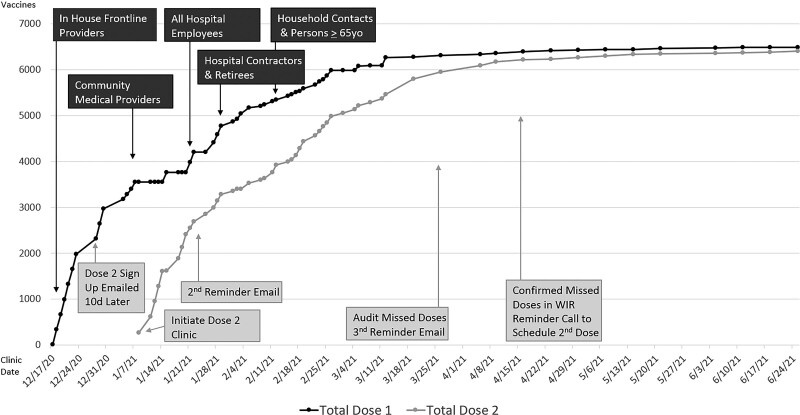
Cumulative number of daily COVID-19 vaccines administered. We administered 6,489 dose 1 and 6,403 dose 2 vaccines, indicated by the black and gray lines. Due to vaccine surplus, we expanded the target audience to include community medical providers, all hospital employees, hospital contractors, retirees, community members over 65 years old, and household contacts of hospital employees. Black PDSA cycles describe the expansion of the vaccine program, and gray PDSA cycles highlight interventions to ensure dose 2 completion.

Figure 3 demonstrates the cumulative number of COVID-19 vaccines administered by clinic day. In total, we administered 6,489 dose 1 and 6,403 dose 2 vaccines, indicated by the black and gray lines. Black PDSA cycles describe the expansion of the vaccine program to other populations, and gray PDSA cycles highlight interventions to ensure dose 2 completion. For example, a second reminder email resulted in most patients completing dose 2, and the third email reminder and phone call improved adherence even further.

Unique to this vaccination effort was the desire to avoid wasted vaccines. Subsequently, only 13 vaccines were wasted (0.1%) in this process. Additionally, all reported side effects have been consistent with the CDC vaccine info sheet.

## DISCUSSION

### Summary

A preimplementation survey suggested that 73% of healthcare personnel at our institution would accept the COVID-19 vaccine immediately or at a later time. The study team surpassed the goal to administer COVID-19 vaccines to all interested Phase 1a staff and successfully vaccinated 83% of hospital/academic center employees (n = 5,231), with over 98% completing the two-dose series. Furthermore, due to the success of the vaccination program, we expanded the program to serve as a community vaccination site providing an additional 1,258 individuals with COVID-19 vaccinations with almost 100% completion of the two-dose series. Finally, the team accomplished this success with limited wasted vaccines.

### Interpretation

Voluntary vaccine acceptance rates among our healthcare workers (83%) surpassed local expectations from our institutional survey (73%) as well as international surveys suggesting 63%–77% of healthcare workers intend to receive the COVID-19 vaccine.^8–10^ We hypothesize that the reasons for high COVID vaccine acceptance rates align with prior studies: high individual perceived risk of disease, prior flu vaccination, and stronger vaccine confidence.^8–10^ Although we did not measure the perceived risk of COVID-19 disease, most of our staff were frontline healthcare workers assumed to be at risk for COVID-19 exposure. Additionally, the majority of staff receive annual influenza vaccine due to institutional mandates.

This article highlights the unique differences of large-scale COVID-19 vaccination programs compared to established influenza vaccine campaigns. We address the challenges of storage and handling, scheduling the two-dose series, and vaccine administration/documentation required for COVID-19 vaccination. Additionally, we summarize the system preparation requirements necessary to provide large-scale vaccine distribution to employees and the community. Although our institution considered utilizing this program to administer COVID-19 booster vaccines, due to high patient volumes, a critical staffing shortage, and the current abundance of COVID-19 vaccination sites in the community, we chose to forgo offering booster vaccines to focus staffing resources on direct patient care and related activities. However, future applications of this model could serve to provide the two-dose series or booster vaccines to employees at any medical or nonmedical institution. Community vaccination sites with limited resources may also benefit from this model and consider partnering with a hospital or academic center to provide COVID-19 vaccines to the local community.

### Future Steps

Given the recent FDA approval of the COVID-19 vaccine and the strong CDC recommendation for vaccine mandates in the healthcare setting, it will be critical to explore vaccine hesitancy in employees and understand how this varies by clinical role, race, and ethnicity. Prior studies demonstrate that providers have the highest level of vaccine uptake; therefore, we need to target vaccine education strategies toward those with the lowest uptake.^11^ Furthermore, vaccine acceptance in patients depends on the strength of the provider/nursing recommendation.^12^ Therefore, there is a need for future studies to determine how provider/nursing acceptance of the COVID-19 vaccine impacts the ability to recommend the vaccine to patients.

### Limitations

We performed this vaccination effort at a single, tertiary care pediatric health system that valued COVID-19 vaccine administration. This institution had a preestablished influenza vaccination program for employees; therefore, these interventions may not be generalizable to institutions without this prior support. At the time of this study, demand for COVID-19 vaccines surpassed the supply; therefore, our team worked meticulously to ensure limited vaccine waste. Given the current surplus of vaccines and CDC recommendations prioritizing never missing an opportunity to vaccinate, the need to monitor waste meticulously may no longer be a necessity.^13^

## CONCLUSIONS

We describe the development and implementation of a COVID-19 vaccination model that successfully vaccinated 83% of employees at an academic pediatric health system and provided vaccines to the surrounding community.

## ACKNOWLEDGMENTS

The authors thank Mark Nimmer for assistance with the study.

## DISCLOSURE

The authors have no financial interest to declare in relation to the content of this article.

## Supplementary Material


