# Supplementary material for: Urgent COVID-19 Vaccination of Healthcare Workers via a Quality Improvement Initiative
Source: Pediatr Qual Saf. 2021 Nov 29;6(6):e532. doi: 10.1097/pq9.0000000000000532 (PMC8615323; doi:10.1097/pq9.0000000000000532)
Supplement: Supplementary file 1 [file pqs-6-e532-s001.pdf]

Supplemental Table 1. Employee Needs Assessment

**Which of the following would help move your intention toward definitely receiving the COVID-19 vaccine?***	**n**	**%**
More information about the vaccine's safety	1,212	70.4%
More information about the vaccine's effectiveness in preventing COVID-19	789	45.8%
More information about the Food and Drug Administration's (FDA's) review of the vaccine	719	41.8%
More general information about the vaccine	680	39.5%
More information about the research to create the vaccine	626	36.4%
I want to hear from other people who get the vaccine before deciding	587	34.1%
Other	468	27.2%
More information about how the vaccine can help control the pandemic in our community	333	19.3%
I would be reassured if some provider leaders received the vaccine first	225	13.1%
Nothing will change my mind -- I will not take the vaccine	217	12.6%
I want other COVID-19 vaccines to be made available before deciding	183	10.6%
I would be reassured if some department leaders received the vaccine first	179	10.4%
I have already had COVID-19 and I don't believe I need the vaccine	110	6.4%

*Answer choices not mutually exclusive
Total Respondents: 1,722
